# Retroperitoneal paraganglioma mimicking a solid pseudopapillary neoplasm of the pancreatic tail and confirmed by percutaneous biopsy: a case report

**DOI:** 10.3389/fonc.2026.1838923

**Published:** 2026-08-04

**Authors:** Jiaqi Song, Wei Sun, Yingzheng Ren, Fuyang Deng, Guixin Zhang

**Affiliations:** Department of Pancreatobiliary Endoscopic Surgery, Second Affiliated Hospital of Dalian Medical University, Dalian, China

**Keywords:** catecholamine crisis, catecholamine detection, contrast-enhanced MRI, differential diagnosis, multidisciplinary team, pancreatic solid pseudopapillary tumor, percutaneous biopsy, retroperitoneal paraganglioma

## Abstract

**Background:**

Retroperitoneal paraganglioma (RPGL) is an uncommon extra-adrenal neuroendocrine tumor. When located adjacent to the pancreas, RPGL may mimic solid pseudopapillary neoplasm of the pancreas (SPN/SPTP) because both lesions may present as large cystic-solid masses with nonspecific symptoms and non-diagnostic conventional tumor markers. Percutaneous biopsy may establish the diagnosis but can be hazardous when RPGL has not been excluded.

**Case presentation:**

A 44-year-old man presented with abdominal distension. He had no history of hypertension, headache, palpitations, or profuse sweating. Laboratory tests, including routine blood tests, liver and kidney function, coagulation profile, and conventional tumor markers, showed no significant abnormalities. Ultrasound, CT, and MRI demonstrated a large multilocular cystic-solid mass in the pancreatic body-tail region, and the initial impression was SPN/SPTP or a pancreatic cystic neoplasm. Plasma or urinary catecholamines/metanephrines and functional imaging were not performed because the lesion was initially considered pancreatic in origin and the patient lacked symptoms of catecholamine excess. Ultrasound-guided 16G core biopsy yielded two tissue cores. Histology and immunohistochemistry showed synaptophysin, chromogranin A, and CD56 positivity with a Ki-67 index of approximately 1%, supporting RPGL. Surgical exploration subsequently confirmed that the tumor arose from the retroperitoneum and compressed, rather than originated from, the pancreas. Tumor manipulation caused marked hemodynamic fluctuation and pulmonary edema, requiring postoperative intensive care. Final pathology confirmed RPGL without definite evidence of metastasis or local malignant invasion.

**Conclusion:**

For large cystic-solid masses in the pancreatic tail or adjacent retroperitoneum, RPGL should remain in the differential diagnosis even when classic catecholamine-related symptoms are absent. Before biopsy, clinicians should prioritize careful assessment of tumor origin, multiphasic contrast-enhanced imaging, and biochemical testing for plasma free or urinary fractionated metanephrines. Biopsy should be selective and should follow multidisciplinary risk assessment with appropriate hemodynamic and interventional contingency planning.

## Introduction

Retroperitoneal paraganglioma is a rare extra-adrenal neuroendocrine tumor arising from paraganglionic chromaffin tissue. Population-based data indicate that sympathetic paraganglioma is uncommon, and recent surgical series continue to describe RPGL as representing approximately 1%-3% of retroperitoneal masses (1, 2). Because RPGLs may occur near the abdominal aorta, inferior vena cava, duodenum, and pancreas, a peripancreatic RPGL can be mistaken for a primary pancreatic tumor.

Solid pseudopapillary neoplasm of the pancreas is also rare, accounting for a small proportion of exocrine and cystic pancreatic tumors. Although SPN/SPTP classically affects young women, it can occur in men and in middle-aged patients (7–10). Both SPN/SPTP and peripancreatic RPGL may present with nonspecific abdominal discomfort or be found incidentally. Routine tumor markers such as CEA, AFP, and CA19–9 are often non-diagnostic. Imaging overlap is also substantial: both may appear as well-demarcated heterogeneous cystic-solid masses with hemorrhagic, necrotic, or degenerative components and variable enhancement (10, 11).

This diagnostic overlap creates a practical dilemma. Tissue diagnosis can be valuable when imaging is inconclusive, but biopsy of an unsuspected RPGL may precipitate hemorrhage or catecholamine-related cardiovascular instability. We report a case of a large RPGL initially interpreted as SPN/SPTP of the pancreatic tail and diagnosed by percutaneous biopsy. The case highlights how absence of classic symptoms can be misleading, why biochemical and imaging evaluation should precede biopsy when RPGL cannot be excluded, and how biopsy should be integrated into a selective multidisciplinary diagnostic pathway rather than used routinely.

## Case presentation

A 44-year-old man was admitted with abdominal distension and discomfort for 3 days. He occasionally experienced abdominal pain after alcohol consumption. He had no history of hypertension or other significant chronic disease. On initial and retrospective review, he denied paroxysmal headache, palpitations, profuse sweating, flushing, or episodic hypertension. On admission, his vital signs were stable, with blood pressure of 115/86 mmHg, heart rate of 59 beats/min, and body temperature of 36.6 degrees C. Physical examination revealed a firm left upper abdominal mass measuring approximately 20 cm x 15 cm with indistinct margins and no obvious tenderness.

Preoperative laboratory tests showed no significant abnormality in complete blood count, liver and kidney function, coagulation profile, or conventional tumor markers. The normal coagulation profile was reviewed before biopsy. Catecholamine or metanephrine measurements were not obtained before biopsy; therefore, the tumor could not be defined as biochemically non-functional. In the revised interpretation, the case is best described as clinically silent or subclinical before surgery rather than definitively non-functional.

**Figure 1:**
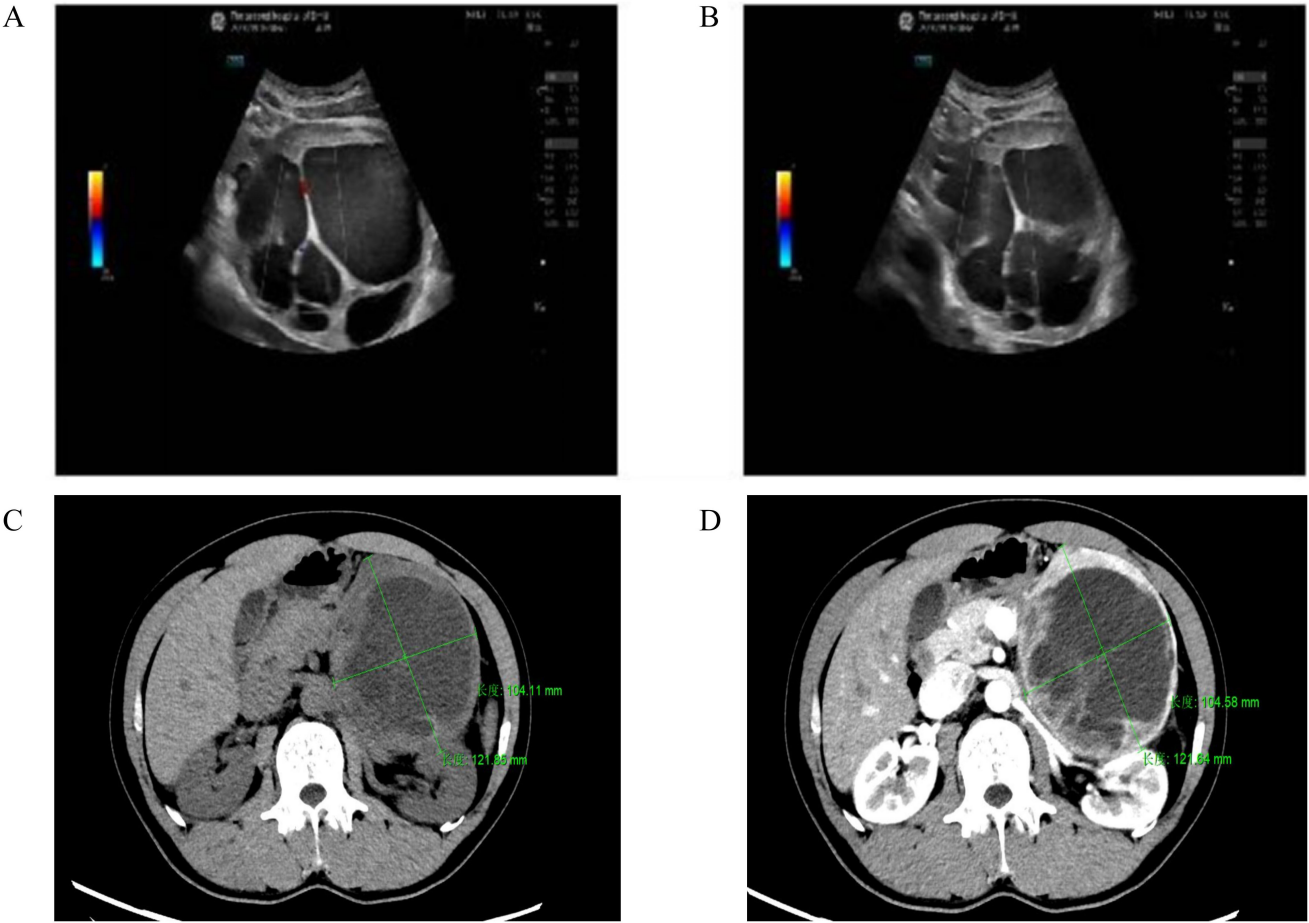
Preoperative imaging. **(A, B)** Color Doppler ultrasonography revealed a predominantly cystic mixed-echo mass located in the pancreatic tail region. **(C, D)** Contrast-enhanced CT demonstrated a large heterogeneous cystic-solid mass with mild enhancement of the cyst wall, septa, and solid components.

Color Doppler ultrasonography demonstrated a predominantly cystic mixed-echo mass in the pancreatic tail region measuring approximately 12.5 cm x 11.1 cm. The lesion had relatively clear borders, coarse septa, poor intracystic acoustic transmission, and no definite blood flow signal within the septa. Non-contrast CT showed a roughly round heterogeneous mass in the pancreatic tail region measuring approximately 12.2 cm x 10.4 cm, with central low density and unevenly thickened cystic walls and septa. Contrast-enhanced CT showed mild enhancement of the cystic wall, septa, and solid components, whereas the cystic components did not enhance (**Figure 1**).

MRI revealed a multiloculated cystic-solid mass in the pancreatic body-tail region, approximately 12.0 cm x 9.6 cm x 14.7 cm. The mass was predominantly hyperintense on T2-weighted imaging and iso- to hypointense on T1-weighted imaging. Diffusion-weighted imaging and ADC maps showed restricted diffusion in the cyst wall, septa, and solid components, but a quantitative ADC value was not documented. These findings, together with normal conventional tumor markers and the apparent relationship to the pancreatic tail, favored SPN/SPTP or a pancreatic cystic neoplasm. Demographically, the patient was not typical for SPN/SPTP because he was a middle-aged man; however, the cystic-solid morphology and apparent pancreatic origin had greater influence on the initial diagnostic impression.

In retrospect, several findings could have raised suspicion for RPGL: the mass was located along the retroperitoneal aspect of the pancreatic tail, compressed adjacent pancreatic tissue, and had close relationships with major retroperitoneal vascular structures. However, the cleavage plane between the lesion and pancreas was not confidently recognized before biopsy. Functional imaging, such as MIBG scintigraphy or somatostatin receptor PET/CT, was not considered during the initial workup because RPGL was not strongly suspected at that time.

**Figure 2:**
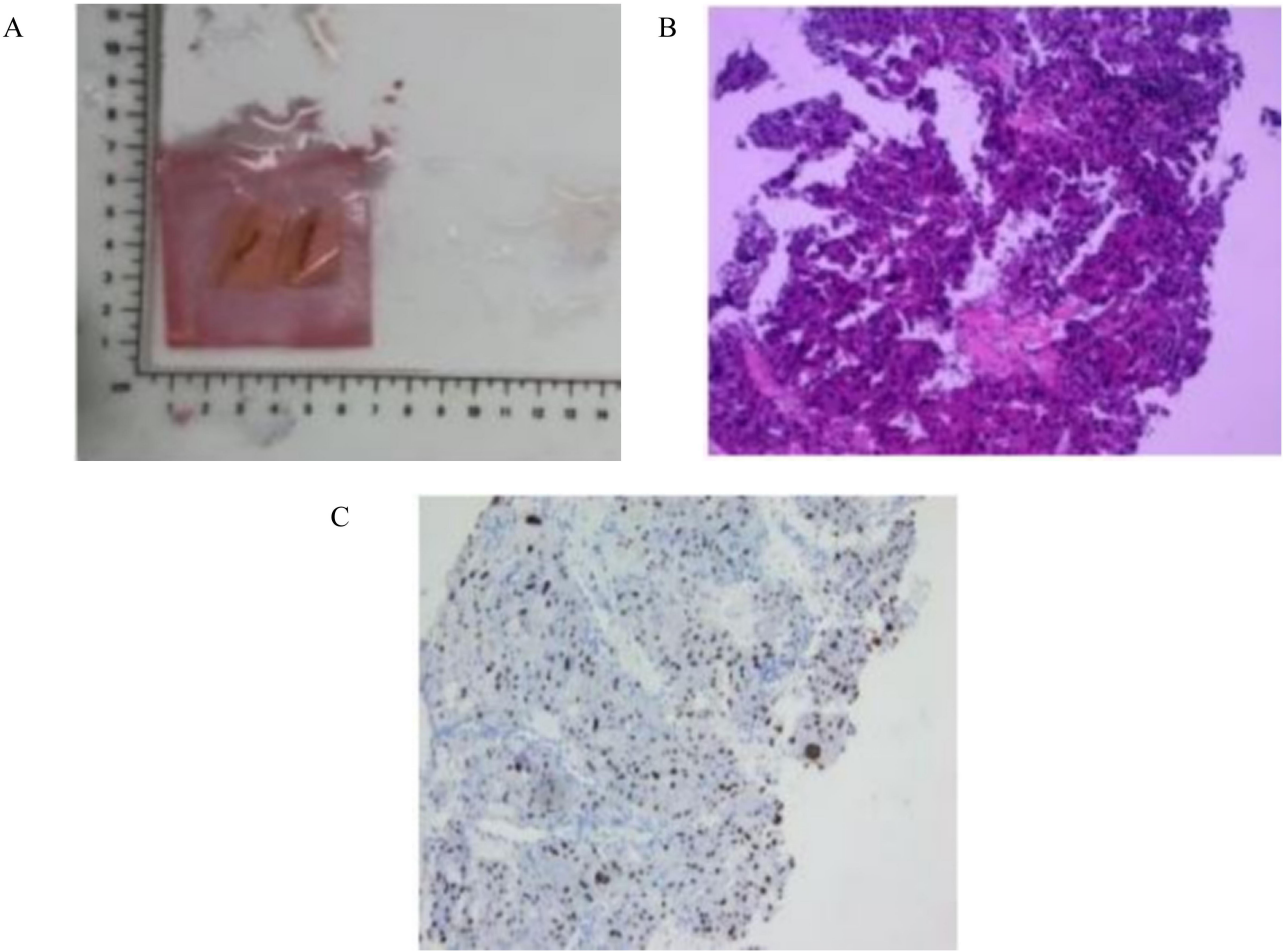
Biopsy specimen and biopsy pathology. **(A)** Ultrasound-guided core biopsy yielded gray-red tissue cores. **(B)** Histology of the ultrasound-guided core biopsy specimen. **(C)** Immunohistochemistry of the Ultrasound-guided core biopsy specimen.

To clarify the nature of the tumor and guide surgical planning, the patient underwent ultrasound-guided percutaneous core needle biopsy. The procedure was performed using a 16G biopsy needle through a single skin entry, and two gray-red tissue cores measuring 1.5-2.0 cm were obtained (**Figure 2**). The route was selected under ultrasound guidance to avoid obvious cystic areas and visible vascular structures. No rapid on-site pathological evaluation was available. Because the mass was initially considered to be a pancreatic tumor rather than RPGL, alpha-blockade, dedicated catecholamine-crisis preparation, and functional imaging were not performed before biopsy. No delayed puncture-site bleeding, hematoma, hypertensive crisis, or other biopsy-related complication was documented.

Biopsy histology showed tumor cells arranged in sheets with abundant cytoplasm and nuclei of variable size and morphology. Immunohistochemistry demonstrated positivity for synaptophysin, chromogranin A, and CD56, with a Ki-67 proliferation index of approximately 1%. These findings supported the diagnosis of RPGL rather than SPN/SPTP.

After biopsy, the case was reviewed by a multidisciplinary team for operative planning. The actual pre-biopsy evaluation did not include a formal MDT discussion; this has been acknowledged as a limitation. Surgical exploration showed that the tumor was located in the retroperitoneal space, growing upward from the inferior margin of the pancreas. It displaced the splenic artery and vein toward the superior margin of the pancreas and compressed adjacent organs without obvious invasion or dense adhesion. These operative findings confirmed that the lesion arose from the retroperitoneal space rather than from the pancreatic parenchyma.

During tumor dissection, the patient developed marked hemodynamic fluctuation, including intermittent tachycardia and sudden blood pressure surges. Coarse bilateral breath sounds and pulmonary edema occurred, consistent with catecholamine release triggered by tumor manipulation. Postoperatively, because of a low oxygenation index and the need for vasoactive support, the patient was transferred immediately to the emergency intensive care unit. He received endotracheal intubation, mechanical ventilation, and vasoactive support, regained consciousness after extubation on postoperative day 3, returned to the general ward on postoperative day 9, and was discharged uneventfully on postoperative day 15.

**Figure 3:**
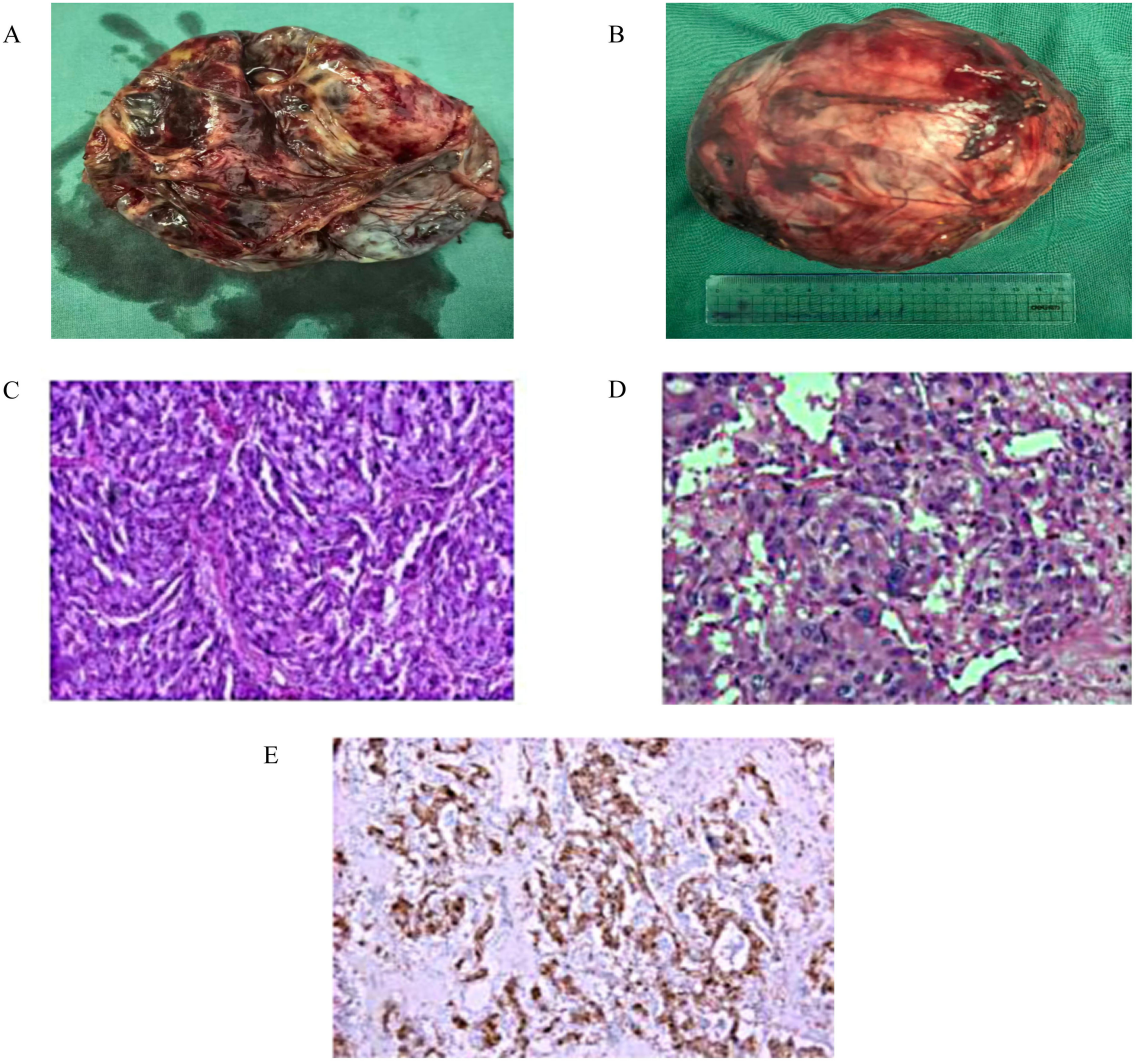
Surgical specimen and postoperative pathology. **(A, B)** The resected tumor showed a reddish-brown surface with prominent vessels. **(C)** Histology of the surgical specimen. **(D, E)** Immunohistochemistry of the surgical specimen.

The resected specimen measured approximately 18 cm x 15 cm and had a reddish-brown surface with multiple varicose vessels (**Figure 3**). Pathology showed tumor cells with abundant amphophilic cytoplasm and pleomorphic nuclei; hemorrhage, necrosis, and edematous degeneration were present. Immunohistochemistry again showed positivity for synaptophysin, chromogranin A, and CD56, with a Ki-67 index of approximately 1%. S100 and SDHB staining were not performed because of specimen and workflow limitations. No definitive intravascular tumor thrombus, perineural invasion, distant metastasis, or gross capsular disruption was identified. Therefore, no definite malignant behavior was confirmed, although RPGLs are considered to have metastatic potential and require long-term surveillance. The GAPP score could not be fully calculated because catecholamine phenotype and SDHB status were unavailable. At the time of revision, postoperative follow-up was limited to approximately 3 months. No symptomatic recurrence had been documented, but surveillance imaging had not yet been completed.

## Discussion

This case is clinically relevant for three reasons. First, it represents a large cystic-solid RPGL in the pancreatic tail region that closely simulated SPN/SPTP, a scenario in which both lesion morphology and apparent site of origin can mislead preoperative diagnosis. Second, the patient had no classic catecholamine-related symptoms, and the tumor was therefore not suspected before biopsy. Third, although biopsy established the diagnosis without immediate complication, subsequent tumor manipulation during surgery produced a catecholamine crisis, demonstrating that clinically silent tumors may still retain secretory potential.

The main preoperative features that favored SPN/SPTP were the large multilocular cystic-solid morphology, apparent pancreatic tail location, mild enhancement of the walls/septa/solid components, T1/T2 signal pattern, restricted diffusion in solid and septal components, and normal routine tumor markers. However, these features are not specific. SPN/SPTP often contains hemorrhagic or cystic components, while large RPGL may also develop cystic degeneration, hemorrhage, and necrosis (10, 13). Therefore, a mass that appears cystic-solid in the pancreatic tail region should not be assumed to be pancreatic unless the organ of origin is clearly established. A comprehensive side-by-side comparison of the clinical, imaging and laboratory features of RPGL and SPN/SPTP is summarized in **Table 1**. This sentence is to be cited in the main text as **Table 1**.

**Table 1:** Practical comparison between RPGL and SPN/SPTP in the differential diagnosis of a cystic-solid pancreatic tail region mass.

Aspect	RPGL	SPN/SPTP	Implication for this case
Typical demographics	Adults of either sex; symptoms depend on secretory status	Predominantly young women, but men can be affected	Male sex was not typical for SPN/SPTP; imaging impression outweighed demographics.
Clinical presentation	May be asymptomatic or present with abdominal discomfort; functional tumors may cause hypertension, headache, palpitations, or sweating	Often asymptomatic or nonspecific abdominal pain/distension	No catecholamine triad was present, which reduced clinical suspicion of RPGL.
Conventional tumor markers	CEA, AFP, and CA19–9 usually non-diagnostic	CEA, AFP, and CA19–9 usually non-diagnostic	Normal markers did not distinguish the two diagnoses.
Anatomical origin	Retroperitoneal; may compress pancreas; fat plane, absent beak sign, and vessel displacement are useful	Arises from pancreatic parenchyma	Retrospectively, the lesion compressed the pancreas and was confirmed intraoperatively as retroperitoneal.
CT/MRI morphology	Solid, cystic-solid, hemorrhagic, or necrotic; often hypervascular but may be heterogeneous	Well-defined encapsulated cystic-solid mass; hemorrhagic/cystic degeneration common	The cystic-solid appearance strongly overlapped with SPN/SPTP.
DWI/ADC	May show restricted diffusion in viable solid components; not specific	Restricted diffusion may occur in solid/septal components; not specific	DWI/ADC restriction was present but did not resolve the differential diagnosis.
Biochemistry	Plasma free or urinary fractionated metanephrines are recommended when PPGL is possible; false-negative results may occur in some nonsecretory tumors	No catecholamine production expected	Testing was not performed pre-biopsy; this was added as a limitation and key lesson.
Functional imaging	MIBG or somatostatin receptor PET/CT may support diagnosis and staging	Not routinely used for SPN/SPTP	Functional imaging was not considered initially because RPGL was not suspected.
Biopsy strategy	Potential risk of hemorrhage and catecholamine crisis; avoid unless selective MDT-based indication	Biopsy may be used selectively when pancreatic diagnosis is uncertain	Biopsy confirmed RPGL but the case supports non-invasive workup before biopsy when RPGL cannot be excluded.

The distinction between pancreatic origin and retroperitoneal compression is particularly important. Reported peripancreatic PGL series emphasize the value of a fat plane between the tumor and pancreas, the absence of a beak sign, and vessel displacement patterns in identifying an extrapancreatic origin (12, 14). In the present case, the pancreatic parenchyma was compressed rather than invaded, and surgery confirmed retroperitoneal origin. Retrospective recognition of this anatomical relationship would have supported biochemical testing and functional imaging before biopsy. Recent published case series and studies describing misdiagnosed Peripancreatic or Retroperitoneal paraganglioma are collated in **Table 2**.

**Table 2:** Selected recent literature on peripancreatic or retroperitoneal paraganglioma mimicking pancreatic/peripancreatic tumors.

Study	Presentation/initial impression	Diagnostic approach	Key lesson
Lanke et al., 2021 (11)	Pancreatic/peripancreatic paraganglioma diagnosed by EUS-FNA; review summarized multiple pancreatic paraganglioma cases	EUS-FNA with immunohistochemistry and MDT interpretation	Morphologic overlap with pancreatic neuroendocrine tumors is substantial; cell block and IHC are essential when biopsy is performed.
Petrelli et al., 2022 (12)	Peripancreatic paraganglioma misdiagnosed as a pancreatic lesion because a cleavage plane was not appreciated	Surgical exploration and pathology; blinded review of imaging/cytology	Failure to define the pancreatic cleavage plane is a major source of misdiagnosis.
Kang et al., 2024 (13)	Subclinical retroperitoneal paraganglioma with nonspecific presentation and imaging	Laparoscopic resection and immunohistochemistry	Clinically silent RPGLs remain diagnostically challenging and require vigilance.
Sunakawa et al., 2024 (14)	Six peripancreatic paragangliomas; some initially resembled peripancreatic/pancreatic masses	Fat-plane assessment, urinary catecholamines, and/or MIBG without routine biopsy	A fat plane, hormone testing, and functional imaging can allow diagnosis and pancreas-sparing surgery.
Shen et al., 2025 (15)	Unexpected RPGL presenting as a retroperitoneal mass with intraoperative hypertensive crisis	Postoperative pathology and perioperative catecholamine testing	Atypical RPGL can be missed preoperatively; invasive monitoring and preparation are important when suspicion exists.
Present case	Large cystic-solid mass in the pancreatic tail region initially diagnosed as SPN/SPTP or pancreatic cystic neoplasm	Percutaneous core biopsy followed by resection pathology	Biopsy can establish diagnosis but should follow biochemical and MDT-based evaluation when RPGL cannot be excluded.

Biochemical testing is central when RPGL is possible. Plasma free metanephrines and 24-hour urinary fractionated metanephrines are recommended screening tests for PPGLs; plasma testing is generally considered highly sensitive, especially in incidentally detected or clinically atypical tumors (3, 4). Nevertheless, normal biochemical results do not absolutely exclude all paragangliomas because some lesions may be nonsecretory, dopamine-predominant, intermittently secreting, or inadequately sampled under suboptimal preanalytical conditions. In the present case, such tests were not performed, and this omission has been explicitly recognized as a limitation.

Functional imaging was also not performed in the initial workup. When biochemical tests are positive or when imaging remains suspicious despite equivocal biochemistry, MIBG scintigraphy or somatostatin receptor PET/CT may help confirm the diagnosis, assess multifocal disease, and plan treatment (6, 14). Genetic evaluation, including SDHB-related assessment, may be considered after diagnosis, particularly for retroperitoneal tumors or patients requiring long-term risk stratification (3). Genetic testing and SDHB immunostaining were not performed in this patient, which limits hereditary risk assessment.

The role of biopsy should therefore be selective. Biopsy can be appropriate when non-invasive evaluation remains inconclusive and the result would substantially alter treatment, but it should not be used as the first diagnostic step when RPGL cannot be excluded. A recent systematic review and individual patient meta-analysis evaluated complications after core needle biopsy in PPGL and supports continued caution, particularly in primary paragangliomas with high catecholamine concentrations (5). In this case, biopsy was justified at the time by the large tumor size, uncertainty about tumor type, need for operative planning, and low clinical suspicion of RPGL. In hindsight, however, the case supports a safer algorithm: first define anatomical origin, then perform biochemical testing and functional imaging when appropriate, and only then consider biopsy after formal MDT review.

If biopsy remains necessary, procedural precautions should include endocrine consultation, consideration of alpha-blockade when RPGL remains possible, continuous hemodynamic monitoring, availability of vasoactive agents, a route that avoids major vessels and cystic/necrotic areas, limited but adequate core sampling from viable solid components, and contingency planning for urgent embolization or intensive care. In the present case, two cores were obtained from solid/septal components under ultrasound guidance, and coagulation parameters were normal. However, no formal pre-biopsy MDT, alpha-blockade, catecholamine testing, or functional imaging was performed. The discussion has been revised to distinguish what was actually performed from what is recommended for future similar cases.

Pathologically, the biopsy and resection specimens supported RPGL by neuroendocrine morphology and positivity for synaptophysin, chromogranin A, and CD56 with a low Ki-67 index. The biopsy specimen did not show features initially mimicking SPN/SPTP; instead, it redirected the diagnosis to RPGL. Malignant potential in paraganglioma cannot be excluded solely by bland histology or low Ki-67 index, because metastatic behavior defines malignancy. In the present patient, there was no documented metastasis, intravascular tumor thrombus, or perineural invasion, but follow-up was short and SDHB/GAPP assessment was incomplete. Long-term imaging and biochemical surveillance are therefore required.

A practical diagnostic algorithm is proposed as follows. For a cystic-solid mass in the pancreatic tail or adjacent retroperitoneum, clinicians should first assess organ of origin using multiphasic CT or contrast-enhanced MRI, paying attention to a fat plane, beak sign, vessel displacement, and relationships to the aorta, inferior vena cava, renal vessels, and splenic vessels. If RPGL cannot be confidently excluded, plasma free metanephrines or 24-hour urinary fractionated metanephrines should be obtained before biopsy. Functional imaging should be considered when biochemical or anatomical features remain suspicious. Biopsy should be avoided when biochemical or imaging findings strongly support RPGL and surgery is feasible. When biopsy is necessary because management would otherwise be uncertain, it should be performed only after MDT review with endocrine, radiology/interventional radiology, surgery, anesthesia, and pathology input.

This report has limitations. Pre-biopsy catecholamine/metanephrine testing, functional imaging, quantitative ADC values, quantitative enhancement values, formal blinded radiology review, SDHB and S100 immunostaining, and genetic testing were not available. Postoperative surveillance was also limited at the time of revision. These limitations were added to avoid overinterpretation of the case and to clarify that the proposed diagnostic pathway is based on the lessons learned from this patient and the recent literature rather than on all elements having been performed prospectively.

## Conclusion

Large cystic-solid masses near the pancreatic tail may represent either pancreatic tumors such as SPN/SPTP or extrapancreatic lesions such as RPGL. Absence of hypertension or the classic catecholamine triad does not reliably exclude RPGL. Percutaneous biopsy can provide a definitive diagnosis, but it should be reserved for selected cases after careful imaging-based localization, biochemical testing, and MDT risk assessment. A balanced approach that prioritizes non-invasive evaluation and selective biopsy can improve diagnostic accuracy while reducing avoidable hemorrhagic and catecholamine-related complications.

## Data Availability

The original contributions presented in the study are included in the article/supplementary material. Further inquiries can be directed to the corresponding author.
